# Overcoming Challenges of Incorporating Higher Tier Data in Ecological Risk Assessments and Risk Management of Pesticides in the United States: Findings and Recommendations from the 2017 Workshop on Regulation and Innovation in Agriculture

**DOI:** 10.1002/ieam.4173

**Published:** 2019-08-07

**Authors:** Steven L Levine, Jeffrey Giddings, Theodore Valenti, George P Cobb, Danesha Seth Carley, Laura L McConnell

**Affiliations:** ^1^ Monsanto Company, Global Regulatory Sciences Chesterfield Missouri USA; ^2^ Current address: Bayer Crop Science, Regulatory Sciences Chesterfield Missouri USA; ^3^ Compliance Services International Lakewood Washington USA; ^4^ Syngenta Crop Protection Greensboro North Carolina USA; ^5^ Baylor University, Department of Environmental Science Waco Texas USA; ^6^ North Carolina State University, Department of Horticultural Science Raleigh North Carolina USA; ^7^ Bayer US, Crop Science, Environmental Chemistry, Research Triangle Park North Carolina

**Keywords:** Ecological risk assessment, Pesticides, Risk management, Higher tier data, Pyrethroid insecticides

## Abstract

Pesticide regulation requires regulatory authorities to assess the potential ecological risk of pesticides submitted for registration, and most risk assessment schemes use a tiered testing and assessment approach. Standardized ecotoxicity tests, environmental fate studies, and exposure models are used at lower tiers and follow well‐defined methods for assessing risk. If a lower tier assessment indicates that the pesticide may pose an ecological risk, higher tier studies using more environmentally realistic conditions or assumptions can be performed to refine the risk assessment and inform risk management options. However, there is limited guidance in the United States on options to refine an assessment and how the data will be incorporated into the risk assessment and risk management processes. To overcome challenges to incorporation of higher tier data into ecological risk assessments and risk management of pesticides, a workshop was held in Raleigh, North Carolina. Attendees included representatives from the United States Environmental Protection Agency, United States Department of Agriculture, National Oceanic and Atmospheric Administration, universities, commodity groups, consultants, nonprofit organizations, and the crop protection industry. Key recommendations emphasized the need for 1) more effective, timely, open communication among registrants, risk assessors, and risk managers earlier in the registration process to identify specific protection goals, address areas of potential concern where higher tier studies or assessments may be required, and if a higher tier study is necessary that there is agreement on study design; 2) minimizing the complexity of study designs while retaining high value to the risk assessment and risk management process; 3) greater transparency regarding critical factors utilized in risk management decisions with clearly defined protection goals that are operational; and 4) retrospective analyses of success–failure learnings on the acceptability of higher tier studies to help inform registrants on how to improve the application of such studies to risk assessments and the risk management process. *Integr Environ Assess Manag* 2019;15:714–725. © 2019 The Authors. *Integrated Environmental Assessment and Management* published by Wiley Periodicals, Inc. on behalf of Society of Environmental Toxicology & Chemistry (SETAC).

## Key Points

- A set of consensus recommendations is provided for principles and practices that can be used to overcome challenges to the incorporation of higher tier data into ecological risk assessments and risk management of pesticides in the United States.
- A summary of 4 different categories of higher tier data is provided.
- Examples of higher tier effects and exposure refinements of ecological risk assessments are described.
- Recommendations include the importance of defining specific protection goals, how to develop fit‐for‐purpose higher tier data, the need for transparency to understand the basis of risk management decisions, and the need to highlight success–failure learnings from the application of higher tier data.

## INTRODUCTION

Overcoming challenges and barriers to incorporating higher tier data into ecological risk assessments (ERAs) and risk management of pesticides was discussed at the 2017 Workshop on Innovation and Regulation in Agriculture held at North Carolina State University on 23–24 October 2017 in Raleigh, North Carolina, USA. A key objective of this workshop was to develop recommendations for principles and practices that can be used to overcome these challenges. Attendees included representatives from the United States Environmental Protection Agency (USEPA), United States Department of Agriculture, National Oceanic and Atmospheric Administration, North American universities, commodity groups, consultants, nonprofit organizations, and the crop protection industry (list in Supplemental Data). Although this workshop was organized by scientists in the United States, with a primary focus on the US pesticide regulatory system, the recommendations from this group are generally applicable in any world area that uses a risk‐based approach and a tier‐based framework for environmental assessments of pesticides.

Workshop participants discussed a range of challenges and potential barriers to the use of higher tier studies as part of the risk characterization in ERAs and to inform risk management decision making. Small group recommendations served as the basis for a whole‐group discussion that created consensus recommendations aimed at addressing the said challenges and barriers. To facilitate dynamic discussion, workshop participants addressed several questions as a starting point:

- What are the characteristics of higher tier studies that make them useful in a risk assessment and for risk management decision making?
- How can the tiered risk assessment process, with respect to the use of higher tier studies, be improved?
- What are the barriers to evaluating and effectively utilizing higher tier studies in regulatory decision making to achieve protection goals?

What are the roles and responsibilities of the risk assessors and risk managers? Risk assessors are a diverse group of professionals who bring a range of multidisciplinary technical expertise to perform a risk assessment. Risk assessors typically follow defined risk assessment guidance and can refine assessments with higher tier data and additional analyses if warranted. The expression “risk manager” is frequently used to represent a decision maker in a regulatory agency—sometimes in a different branch than the risk assessors—who has legal authority to protect or manage a resource. Therefore, risk managers receive the greatest value from an ERA when it is based on environmental protection goals that are operational. For protection goals to be operational, they must be specific, clearly stating what to protect, the level of protection, where to protect it, and over what time period. Risk managers within regulatory agencies have the responsibility and authority to act or require action to mitigate an identified risk (USEPA 1998). However, there is little specific guidance on integrating higher tier data and analyses into ecological risk assessments (ERAs) for pesticides in the United States, and therefore good communication is needed between the risk assessor and risk manager to help the risk manager reach a defensible regulatory decision.

The present report provides brief background on the concepts associated with a tiered approach for ERA and presents representative case study examples of higher tier effects and exposure refinements that have been used by regulatory authorities. Major themes emerging from the workshop are presented as a series of consensus recommendations.

## TIERED RISK ASSESSMENT APPROACH

Registration of a pesticide requires an assessment of potential ecological risk, and the utility of applying tiered ERAs for evaluation of pesticides has long been recognized (Baker et al. 1994; USEPA 1998; Campbell et al. 1998; ECOFRAM 1999). The term “lower tier” is synonymous with “screening level,” and such assessments are typically conservative and conducted using a limited amount of information and basic analysis tools. However, screening‐level risk assessments can serve a valuable purpose either by identifying pesticides and their uses that require no further evaluation or by highlighting specific concerns (e.g., vulnerable taxa or regions, specific use patterns) for further evaluation.

With progression through the tiers, estimates of exposure and effects become more environmentally realistic and uncertainty is reduced through the acquisition of more relevant data. If lower tier assessments indicate that a pesticide may pose a risk to the environment, additional data generation or more advanced quantitative analysis can provide greater insight into the likelihood and magnitude of potential ecological effects. However, the analysis phase of the risk assessment can also consider qualitative information in a weight‐of‐evidence approach. Only after the collective findings of the risk assessment are considered can a decision be made as to whether higher tier testing is required and/or risk management is necessary. Therefore, the need for additional higher tier ecological data may be supplanted by mitigating risks via changes to label requirements, implementing best practices, and product stewardship.

Advancing to a higher tier ERA does not always negate conclusions of inferred risk, but rather may provide additional support and certainty confirming such conclusions. Given that ERA is an iterative process, as more insight is gained with advancement to higher tiers, risk assessors may revisit conceptual models or assumptions utilized during the screening‐level evaluations. The tiered system as a whole should result in protective decisions that are consistent with specific protection goals. In Europe, higher tiers are also used to verify the protective nature of lower tier assessment methods (van Wijngaarden et al. 2005; Solomon et al. 2008; EFSA 2013; Brock et al. 2016).

Although higher tier methodologies typically provide data to address the assumptions and simplifications inherent in lower tier risk assessments, the design, conduct, and acceptance of these approaches can pose specific challenges. For example, in mesocosm studies, adequate replication may be difficult to achieve and appropriate control treatments may be difficult to devise, leading to low statistical power for detecting pesticide effects. To avoid such problems, higher tier studies should be conducted only when appropriate methodologies (e.g., aquatic mesocosm studies following guidelines) are available and their value to the risk management decision is confirmed by a regulatory authority. The nature and scope of the studies that could be performed will depend on several factors, including the results of lower tier studies, the environmental protection goals and related assessment endpoints, the environmental fate of the pesticide, and the use pattern of the pesticide. It is also important to recognize that science continually evolves, and consequently, improved approaches to conduct environmental assessments will replace older approaches.

## TYPES OF HIGHER TIER DATA

Readers of the present manuscript may question why a workshop addressing challenges with incorporating higher tier data into ERA is needed, given that the current USEPA ERA paradigm for pesticides allows for the flexibility to incorporate higher tier data. For instance, the new USEPA “Guidance for Assessing Pesticide Risks to Bees” outlines a tiered testing and assessment framework that is linked to specific measurement endpoints, assessment endpoints, and protection goals (USEPA et al. 2014). However, even with this new comprehensive testing and assessment guidance, standardizing protocols for conducting higher tier semifield and field effects tests continue to evolve. Consequently, the need remains for discussion with regulatory agencies to align on key aspects of nonstandard protocols. Without this dialogue, registrants run the risk that their study may not have the appropriate design and analysis to support a higher tier assessment.

For the purposes of the workshop and this manuscript, higher tier data can be defined as information that goes beyond standardized data requirements to inform regulatory risk assessments and/or risk management decisions. This definition expands beyond the conventional view that higher tier data are derived only from studies, but also includes other sources of scientifically relevant information that may quantitatively or qualitatively refine risk assessments to support risk management decisions. Four broad categories of higher tier data were emphasized during the workshop: experimentally derived data, model‐generated data, compiled data, and data developed via analysis (Table 1). A list of general examples under each category is provided to illustrate the types of data that are potentially useful for refining risk assessments or informing risk management decisions.

**Table 1:** Summary of different categories of higher tier data and examples that may be useful for refining ecological risk assessments and/or informing the risk management decision concerning pesticides

Broad categories of higher tier data	General examples
Experimentally derived	Laboratory bioassays performed with additional species (perhaps nonguideline), life stages, conditions (i.e., pulsed‐dosed), etc
	Mesocosm or microcosm studies examining the fate and/or effects
	Off‐field transport studies examining transport via air or particulates
	Repeating dated guideline studies using emerging technologies (e.g., partitioning studies using solid phase microextraction)
	Studies focused on addressing specific assumptions in current models (e.g., avian dermal absorption, dietary residues).Toxicokinetic studies exploring adsorption, distribution, metabolism, and excretion
Model generated	Additional PWC model simulations using refined inputs to define exposure
	Development of scenarios alternative to a farm pond (e.g., flowing waterbodies, estuarine or marine systems)
	Establishment of broader level landscape exposure modeling (i.e., watershed).Creation of new models to address specific assumptions and/or accommodate for data limitation
	Development of toxicokinetic–toxicodynamic models
	Species population modeling
Information compiled	Wildlife surveys
	Environmental monitoring databases
	Weight‐of‐evidence approaches to inform hazard endpoints
	Collection of more detailed regional use information concerning rates and timings
Developed via analysis	Probabilistic assessments (e.g., species sensitivity distributions and development of joint probability curves)
	Establishment of mechanistic or adverse outcome pathways
	Species range and use area proximity analysis.Incorporation of advanced statistics.Development of refined conceptual models

PWC = Pesticide in Water Calculator.

Experimentally derived higher tier data, as implied in the category name, involve generation of information as part of a study that can be used to support an ERA. It is important to note that while higher tier data are often associated with larger and more complex experiments designed to narrow the gap between laboratory and real‐world conditions (e.g., microcosms, field studies), increased complexity is not always needed. Rather, experimentally derived higher tier data can range in scope and scale from very simple to extremely complex, and the level of complexity is based on the purpose, questions, and concerns for which additional information is needed. In some cases, a basic but focused laboratory experiment may be sufficient to refine an ERA or inform a risk management decision. For example, testing under nonstandard conditions may be used to consider how abiotic factors (such as temperature) may modify chemical toxicity. In other instances, an expansive field study at numerous sites conducted over multiple field seasons may be deemed necessary.

Model‐generated higher tier data are developed by using computational tools that leverage available information. These data may range from simple calculations to refining a specific assumption of an existing regulatory model, to the development of an entirely new model. The intended use of a model will affect the level of precision and validation required for it to be deemed useful for quantitatively refining an ERA or informing a risk management decision. For example, complex models designed to understand spatial–temporal distinctions in fate and behavior of a pesticide across a large diverse geography, and to encompass different use scenarios, would require far more development and validation than a model meant to simulate one regional scenario for a single crop.

Compiling data for a higher tier ERA involves the collection, analysis, and interpretation of existing information to inform an ERA or risk management decision. Like all categories of higher tier data, the purpose, questions, and concerns prompting the need for compiled data should be established a priori in consultation with regulators so that relevant information is collected. Data compilation should consider appropriate experimental design, methods, quality control measures, inclusion and exclusion criteria, and data analysis methods. In addition, an explicit analysis and characterization of the uncertainty in a data set itself is an important step. For example, evaluating the timing and frequency of sampling will influence the level of confidence and uncertainty in being able to use measured data quantitatively. Merely developing a database (e.g., water monitoring) without a clear link to how the information is collected and can be reliably used is unlikely to prove useful for refining an ERA or informing a risk management decision.

Higher tier data developed by analysis are based on procedures that allow the best available information to be interpreted or applied to refine an ERA or inform a risk management decision. As an example, in 2013 the National Research Council (NRC) identified the need to better characterize risk in endangered species assessments and recommended that screening‐level risk assessments based on deterministic risk quotients (RQs) should be followed by refined probabilistic assessments (NRC 2013). Similarly, in their recent report on future biotechnology products, the NRC also stressed the need to incorporate probabilistic risk assessment procedures (NRC 2017). The rationale for this recommendation is that RQs do not estimate risk (i.e., the probability of an adverse effect). However, probabilistic approaches can provide risk managers with a risk estimate that reflects the probability of exposure to a range of pesticide concentrations and the magnitude of an adverse effect (if any) resulting from such exposure. When protection goals are defined in probabilistic terms, probabilistic risk estimates can be used by risk managers to inform regulatory decision making and potentially to be used as a risk communication tool (e.g., there is a 1% chance of terrestrial nontarget plant communities being affected above their no effect level from applications of herbicide “X” when label requirements are followed). In addition, qualitative data can be effectively leveraged to refine assessments using a weight‐of‐evidence approach. Examples of this could include information describing an organism's feeding ecology, habitat requirements, development and resource utilization timing, and other information that can be used in a weight‐of‐evidence assessment.

## ENSURING HIGHER TIER DATA IS “FIT FOR PURPOSE”

Tiered testing and assessment schemes have been developed to be a flexible framework to address changing testing and assessment needs and new hypotheses that may arise during problem formulation or as the outcome of lower tier testing. Therefore, a tiered framework offers many advantages in terms of supporting assessments that examine potential effects of pesticides. A common goal among data generators, sponsors, reviewers, and users of the data should ideally be to design an appropriate higher tier study to satisfy the needs of the risk assessor or risk manager within the bounds of current capability and without imposing unnecessary regulatory burden on registrants. An advantage for conducting some higher tier studies (e.g., mesocosms) is that endpoints can be more relevant to actual environmental exposure, if properly designed, because factors that influence fate and exposure can be incorporated directly into the test system. Consequently, such studies should provide more confidence when predicting actual effects in the environment. In addition, tests can be designed to assess effects for a specific taxon that may be at risk, and postexposure recovery can be assessed for both individuals and populations (EFSA 2016). However, there are challenges to conducting higher tier tests. For example, standardized higher tier methodologies are generally not available, and there can be disagreements over the relevance of different test designs and interpretation of outcomes. Therefore, having a discussion between registrants and regulators to agree on the goals, technical approach, data evaluation, and ultimate utilization of the information is a critical step in the process to gain acceptance for higher tier assessments.

In 2006, the USEPA published guidance that can be used prospectively in planning and retrospectively in an evaluation against performance and acceptance criteria for environmental testing that will inform defensible decisions using relevant and reliable data (USEPA 2006). The data quality evaluation process offers a robust approach to ensure that data are of sufficient quality to support the goals of the study and the ERA. An important goal of the data quality process is to have an efficient and effective expenditure of resources and for the process to be used to develop an agreement on the type and quality of data needed to meet a study goal. For example, to achieve a specific protection goal, there should be agreement with the regulatory authority on the specific assessment endpoints, the indicators of effect, and the measurement endpoints. If guidance or guidelines are not available to address a specific assessment endpoint with a higher tier study, it becomes even more important to have a discussion with the regulatory authority to reach consensus on the design, analysis, interpretation, and application of the data to the ERA.

**Figure 1:**
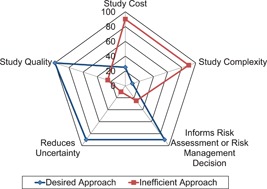
A conceptual scheme illustrating the interplay of factors that should be considered prior to generation of higher tier data.

Focused approaches that answer a specific concern in a risk assessment can provide useful data that reduce uncertainties of potential effects under environmentally realistic exposure conditions. The desired approach is to minimize study complexity and study cost while maximizing study quality and value to the risk assessment as shown in the conceptual diagram, Figure 1. An inefficient approach is to introduce unnecessary complexity and study cost. Presently, there is limited guidance in the USA and some other world areas on the design and interpretation of higher tier approaches and how they can be easily incorporated into a risk assessment that informs regulatory decision making. Although there has been progress on the design and application of higher tier testing in recent decades (EFSA 2013; Overmyer et al. 2018), there is still a need to achieve better consistency in the design, analysis, and interpretation of studies. Objectives of higher tier studies will vary with the questions being addressed. For example, higher tier studies may be performed to characterize the effects of continuous versus noncontinuous exposure; assess effects on sensitive life stages; focus on a specific taxon of concern; determine the potential for organisms, populations, and communities to recover after exposure; or determine indirect effects of compounds on biological communities.

## EXAMPLES OF HIGHER TIER EFFECTS AND EXPOSURE REFINEMENTS FOR ECOLOGICAL RISK ASSESSMENTS

### Higher tier effects refinement

#### Population modeling

Population modeling provides a powerful tool in evaluating and managing ecological risks from pesticides. Basic population models have existed for more than half a century (Barnthouse 1992). Regulators have recognized the need to incorporate population models into risk assessment and management processes since the 1990s (Barton 1994), and this prompted a Society of Environmental Toxicology and Chemistry (SETAC) Workshop on Population Ecology and Wildlife Toxicology of Agricultural Pesticide Use: A Modeling Initiative for Avian Species, held at Kiawah Island, South Carolina, USA on 22–27 July 1990 (Kendall and Lacher 1993). These early models were beginning to estimate effects on terrestrial (Lari et al. 1994; Madrigal et al. 1996) and aquatic organisms (Connolly 1985; Barnthouse 1992). Increased computational power and agreement on probabilistic risk paradigms allowed regional scale assessments to emerge (Solomon et al. 1996, 2001; Purucker et al. 2007). Recent models may incorporate more refined predictions of exposures and effects across landscapes (Schmolke et al. 2010; Dixon 2012; Bartell et al. 2013; Kohler and Triebskorn 2013; Van den Brink 2013; Wang 2013; Focks et al. 2014; Topping et al. 2015; Dohmen et al. 2016; Hilbers et al. 2018). These approaches allow prediction of population exposures to pesticides and resultant responses without having to conduct painstaking multiyear field assessments of population dynamics. Population models also indicate the organisms that may be at greatest risk and the parameters that may reduce those risks. New approaches are emerging that also include mechanistic modeling of effect to evaluate multiple chemical or other contributing stressors (Hanson and Stark 2012; Gergs et al. 2016). Including these techniques along with field validation of model results may expedite the review process while allowing continued improvement of risk assessment and management. In fact, population modeling provides the basis for the combined Terrestrial Investigation Model/Markov Chain Nest Productivity Model (Etterson et al. 2017), which is an approach that the USEPA now suggests for assessing terrestrial ecological risks.

Before a population model can be used to support a regulatory assessment, several factors need a detailed evaluation and discussion with the regulators. These factors include the model's realism, relevance, flexibility, treatment of uncertainty, prior regulatory acceptance, and credibility (Pastorok and Akcakaya 2002). The realism of the model should be assessed, reviewing whether the assumptions are realistic with respect to the ecology of the species. The relevance of the model should be assessed by how related the model results are to the population endpoints that are used in the ERA. The model also should have sufficient flexibility for use at different spatial scales and temporal distributions. The model should also be able to incorporate uncertainties to an adequate level to inform regulatory decision making. Finally, the population model or approach should have credibility in the literature and a prior degree of regulatory acceptance (EFSA 2014; Grimm et al. 2014; Hommen et al. 2016). Without having a moderate to high level of confidence for each of these criteria, acceptance of the model in a regulatory environment will be challenging.

#### Dietary toxicity studies

For avian and wild mammal assessments, acute toxicity is typically evaluated by gavage with a single bolus dose. However, with wildlife exposure to pesticides, a continuous dietary exposure that allows time for metabolism is often the more realistic exposure over a single high‐dose administration by gavage. Therefore, an assessment of the effects of acute dietary exposure can be evaluated in continuous no‐choice feeding assays and used in a weight‐of‐evidence context (USEPA 2004). Dose can be estimated by measuring food consumption and accounting for wastage, which may be important for some species. In addition, the calculation of cumulative dose could be confounded if birds are not individually housed. However, for species like quail that are covey species, individual housing at the young age required for a short‐term dietary study will result in extreme stress and potentially death. Consequently, the latter 2 points should be discussed with the regulators before applying this test type as a refinement to a risk assessment. These dietary exposures can be expressed as a daily dose or a cumulative dose over the feeding period. An area in which this approach has been utilized is with testing of surfactants. Surfactants, as indicated by their name, are surface‐active molecules. At unrealistic supraphysiological doses, administered primarily by gavage, these compounds can cause severe gastrointestinal irritation that would not occur under a normal feeding situation. For this reason, USEPA allowed continuous dietary feeding exposures with Organisation for Economic Co‐operation and Development (OECD) 422 guideline studies, rather than daily dosing by gavage, for surfactants during their assessment for a tolerance exemption (USEPA 2009). Allowing dietary exposure, for some classes of substances, can build more realism into the assessment and not confound the results.

#### Species sensitivity distributions for pyrethroid insecticides

Toxicity endpoints used in screening‐level assessments typically are based on the most sensitive of a small number of standard tested species in each taxonomic group. This approach is prudent when data are limited because it is highly likely to be protective of the most sensitive species in any habitat. However, when more data are available, the effects assessment can be refined to focus on other questions and uncertainties: How does the pesticide affect the range of species in a community (not only the most sensitive species)? What is the likelihood of an effect on a particular untested species? Analysis of species sensitivity distributions can be highly informative when data are plentiful and enables a probabilistic approach that addresses risk questions in terms of magnitude and likelihood of effect (ECOFRAM 1999; Posthuma et al. 2001).

The toxicity database for pyrethroids is especially rich, with data for more than 300 aquatic species (Giddings et al. 2016). Examination of the entire database shows that the freshwater amphipod *Hyalella azteca* (a standard test species) is the most sensitive tested species for every pyrethroid active ingredient. Overall, 95% of the insect and crustacean species are at least 5 times less sensitive than *H. azteca*. Fish and mollusks are much less sensitive than insects and crustaceans. This information provides important context for interpretation of the toxicity data used in the screening‐level assessment.

### Higher tier exposure refinements

#### Precision planting of treated seed

Advancements in precision planting technology can reduce environmental exposures from seed treatments to terrestrial and aquatic organisms. In this scenario, exposure of birds and wild mammals can be greatly reduced by achieving high incorporation efficiency for treated seeds. Seed drilling technology and incorporation efficiency have significantly advanced over the past decades to nominally achieve ≥99% incorporation efficiency for some crops such as corn (USEPA 2016a). When bird and wild mammal assessments are refined to include precision planting, there is a significant reduction in the exposure. The USEPA has leveraged this refinement as a risk management option in registration decisions (USEPA 2010, 2016b). In addition, precision planting technology allows treated seeds to be planted at minimum required depths, which can mitigate pesticide runoff to aquatic environments. Reduction in runoff will vary with planting depth and the pesticides organic carbon–soil adsorption coefficient (*K*_OC_). For substances with high *K*_OC_ values (i.e., >2000) that are minimally buried at a 0.5‐inch depth, modeled runoff under most scenarios can generally be reduced by >99% and minimal runoff would be predicted at a planting depth ≥1 inch (USEPA 2016a). Presently, seed spills, which could confound assessments based on high incorporation rates, are addressed on product labels. To accurately characterize seed incorporation and risks to seed spillage, an assessment of seed spillage during transport and planter loading may be an important refinement for a risk assessment. Therefore, to improve the acceptability of data that support these refinements for a given crop type, and to address the issue of seed spillage more directly, consultation with regulators is recommended to develop the higher tier data that support these risk refinements to aquatic and terrestrial assessments.

#### Improved measurements of partition coefficients for pyrethroid insecticides

Pyrethroid insecticides are characterized by their extreme hydrophobicity and resultant tendency to partition to particulate and dissolved organic matter. Only freely dissolved pyrethroids are readily bioavailable to most aquatic organisms; bound pyrethroids do not cause toxicity (Day 1991; Di Toro et al. 1991; Hamer et al. 1999; Maund et al. 2002; Yang et al. 2006; Hunter et al. 2008). Accurate estimates of freely dissolved pyrethroid concentrations are therefore critical in ERA as well as in interpretation of surface water monitoring data. Whereas standard analytical methods for pyrethroids measure total pyrethroid (bound plus freely dissolved), concentrations of freely dissolved pyrethroid alone can be measured using techniques such as solid phase microextraction (SPME) (Bondarenko et al. 2008; Hunter et al. 2008). The Pyrethroid Working Group (PWG) used SPME to measure *K*_OC_ and dissolved organic C partition coefficients (*K*_DOC_) for 9 pyrethroid active ingredients, using natural and artificial sediments widely used in sediment toxicity testing (T Xu, Bayer Crop Science, personal communication). These partition coefficients enable more accurate prediction of freely dissolved concentrations with standard regulatory environmental fate models (e.g., Pesticide in Water Calculator, USEPA 2016c) and more accurate estimation of freely dissolved concentrations in surface waters.

#### Pathway identification study with pyrethroid insecticides

Outdoor residential uses of pyrethroids are a major source of inputs to urban water bodies (USEPA 2016d). The PWG constructed a full‐scale physical representation of a typical residential environment, with home fronts, garages, driveways, and lawns, for studies measuring off‐target pyrethroid transport from different residential use patterns (Davidson et al. 2014). The house plots were treated according to current labels as well as historic practices in a study that extended across several seasons. The results identified specific impervious surfaces and use patterns that contributed the most to pyrethroid runoff in urban environments. The PWG used the data from this study to refine the aquatic exposure model for outdoor residential uses (Jackson and Winchell 2011; Giddings et al. 2017). To achieve maximal impact from a study of this nature, the design and exposure scenarios should be based on an agreed‐upon approach among registrants, study designers, risk assessors, and risk managers.

#### Exposure modeling uncertainty analysis

The observed discrepancy between modeled and measured pyrethroid exposure concentrations led PWG to an exploration of model uncertainty (Giddings et al. 2017). Exposure modeling deals with uncertainties and natural variability by making assumptions, explicit or implicit. In the screening level, assumptions are intentionally conservative, but the cumulative effect of these assumptions has rarely been quantified. The PWG identified more than 30 key sources of uncertainty associated with the exposure scenarios, model algorithms, and model input values (P Hendley, Phasera Ltd., personal communication). They tested the effect of these uncertainties, individually and in combination, on the model output. When the screening‐level model parameters were varied based on the likely range of real‐world values, estimated exposure concentrations (EECs) were reduced. The combined effect of the uncertainties (based on EEC reductions) ranged from 5× to 380× across different crop scenarios. If the model was adjusted for Percent Cropped Area, the EEC reductions ranged from 11× to nearly 10 000×. Nearly all the level of concern exceedances noted in the screening‐level assessment were eliminated when even a small subset of these uncertainties was considered.

### Higher tier exposure and effects refinement

#### Drift to nontarget plants

In guideline vegetative vigor studies (OCSPP 850.4150; USEPA 2012), the foliage of exposed test plants is completely saturated with solutions containing test material over a gradient of concentrations. Such exposures are analogous to what would occur for an in‐field target weed application (USEPA 2012). However, protection goals are generally not focused on protecting plants in‐field, but rather those located off‐field. Unlike the standard guideline studies in which plants receive uniform direct applications, nontarget terrestrial plants located off‐field generally experience exposure via airborne drift in which droplets are dispersed and exposure is determined by not only the distance from field but also numerous other factors that affect the interception and deposition on plant surfaces (Brain et al. 2017). Notably, these latter factors are not considered by the screening‐level drift simulation model AgDRIFT (Teske et al. 2002) but are captured by the field‐based drift exposure and biological effects study conducted by Brain et al. (2017). In this type of study, plants are exposed at different distances downwind from a spray boom, and then transported back to the laboratory for biological evaluations consistent with those outlined in OCSPP 850.4150 (USEPA 2012).

#### Effect of sediment on aquatic toxicity

Once a compound enters an aquatic environment, its distribution will depend on several factors, such as the potential for abiotic and biotic degradation and the partitioning behavior of the compound to suspended particles or sediment. Higher tier studies can investigate the incorporation of one or more of these processes into intermediate‐tier laboratory or higher tier field studies. For example, by adding sediment to the standard test system, degradation and adsorption processes occurring in the environment can be simulated (Hamer et al. 1992). Studies with pyrethroids have demonstrated that the presence of sediment in the test system significantly reduces the observed effects of a pyrethroid to pelagic aquatic animals (Mancini 1983; Clark et al. 1989; Maund et al. 1998; Shillabeer et al. 2000). This reduction in toxicity reflects that a small fraction of the introduced pyrethroid is available in the water column due to partitioning of the compound from the aqueous phase to sediment. While reducing exposure to animals in the water column, partitioning may increase exposure to benthic animals.

#### Effects of time‐varying exposure

Toxicity studies can be performed with varying exposure intervals to determine the time to effect and the magnitude of effect for different exposure regimes. For example, *Gammarus pulex*, freshwater amphipod crustaceans, were exposed to the pyrethroid lambda‐cyhalothrin for 1, 3, 6, 12, or 96 h, and after exposure organisms were transferred to clean water for 96 h and observations were taken (Maund et al. 1998). There was a significant relationship between decreased effects with decreased duration of exposure, and information of this nature can be leveraged to refine an assessment based on exposure profiles. In addition, time‐variable exposures can be addressed, and uncertainties reduced, by toxicokinetic and toxicodynamic modeling approaches (Gergs et al. 2016).

#### Mesocosm studies

Mesocosms (and their smaller analogs, microcosms) are physical models of communities and ecosystems, used to study the fate and effects of pesticides and other chemicals on populations and communities under quasirealistic exposure conditions. Properly designed and conducted, mesocosms can provide information on the responses of a wide range of taxa, many of which are difficult or impossible to test under standard conditions (Giddings et al. 2001, 2002; Arts et al. 2006). Indirect effects of chemicals, and ecological recovery from chemical effects, can also be observed. Mesocosms also allow detailed investigation of pesticide fate. Furthermore, such studies can be designed to examine effects of real‐world exposure scenarios by simulating chemographs from monitoring sites (King et al. 2016). Mesocosms are typically used in the later stages of an ERA, either to confirm the conclusions of the lower tiers or to investigate a specific issue of concern.

The utility of such studies for a regulatory assessment is highly dependent upon 4 things. First, an agreed‐upon exposure scenario. Second, an agreed‐upon minimum power of the study to support either univariate, multivariate, or both types of statistical evaluations. Third, agreement on the types of endpoints that will comprise the basis for evaluation. Fourth, inclusion criteria that will help to assess the performance of the study, which will help to support its use in regulatory decision making (EFSA 2013).

## DISCUSSION AND CONCLUSIONS FROM CONSENSUS RECOMMENDATIONS

Higher tier approaches can have several advantages over standard environmental studies if they are designed correctly and are fit for purpose to support the risk assessment or risk management options. Some important advantages of higher tier studies are that they can provide more realistic measures of exposure, more confident predictions of actual effects in the environment, and additional relevant endpoints for the risk assessment, such as the potential for recovery of individuals and populations.

Challenges in the application of higher tier studies in regulatory decision making can involve aspects of study design, analysis, and interpretation, as well as their application to assessment endpoints for the risk assessment and how they inform the risk management process. Several themes emerged throughout the workshop discussions that became consensus recommendations. Broadly, these recommendations address the need for improved communication on higher tier approaches between registrants and regulators before studies are initiated; emphasizing that higher tier studies do not need to be complex to provide high value to the regulatory process; the idea that all stakeholders would benefit from improved transparency relative to risk–benefit assessments which then support risk management decisions; and the suggestion of a learning opportunity based on successes and failures of higher tier testing and risk mitigation approaches. A summary of the workshop recommendations is provided in Table 2. Each recommendation is expanded on in the following paragraphs and will help to overcome current challenges to incorporate higher tier data in ERAs and risk management of pesticides in the United States.

**Table 2:** Consensus recommendations from workshop participants

Consensus recommendations
1) More effective and open communication among registrants, USEPA risk assessors, and risk managers is needed earlier in the registration and registration review processes to clarify specific protection goals, assessment endpoints, and measurement endpoints to address areas of concern. a) Registrants should confer with USEPA early and often to align regulatory goals with assessment objectives. b) Specific protection goals should be clear, concise, transparent, and actionable with specific assessment and measurement endpoints established in advance of initiating a higher tier study.
2) Study design should be carefully considered to minimize complexity and to provide high value to the risk assessment and risk management process. a) When a screening‐level assessment indicates potential risk, risk assessors should consider incorporating higher tier data and assessment procedures using a stepwise process (simple → complex) to refine the risk characterization and better inform risk management decisions. b) When additional data are required, USEPA and registrants should prioritize studies that will provide highly relevant findings and address the most significant issues of concern. c) Methods for higher tier studies should be formalized and implemented in guidance documents. Protocols should provide well‐defined decision criteria to assess the performance and acceptability of the study and to meet USEPA's Guidance on Systematic Planning Using the Data Quality Objective Process. d) Development of standard evaluation procedures (SEPs) is recommended for commonly requested higher tier studies. e) When providing higher tier studies as supplementary information, registrants should provide a clear and concise executive summary that describes how the supplementary information informs risk assessment and/or risk management decisions.
3) Greater transparency is needed to understand the basis for risk versus benefit decision making and other critical inputs that factor into risk management decisions. a) When a risk management decision is made, it is recommended that regulatory authorities provide a summary of all data and procedures that were considered, utilized, and/or excluded in the decision‐making process. b) A transparent and well‐documented process is required to evaluate the relevance and reliability of existing higher tier data from the literature.
4) Retrospective analyses of success–failure learnings on the acceptability of higher tier studies would be a valuable exercise to inform registrants on how to improve the generation of higher tier studies. a) A retrospective review is recommended to provide cases where higher tier studies were conducted and clearly helped to inform risk assessment or risk management decisions. b) A postmortem meeting on the pyrethroid risk assessment and risk management process is recommended to identify what worked and where the process could have been improved regarding acceptance or rejection of higher tier data.

USEPA = United States Environmental Protection Agency.

The first consensus recommendation from the group called for more effective, timely, open communication among registrants and governmental risk assessors and risk managers throughout the registration review processes. Early communications are vital to understanding proposed uses and rates for products so that the correct exposure scenarios can be considered, the scope and nature of data required to support the ERA can be defined, and the objectives of the assessment can be agreed upon. For example, a dialogue may be needed to clarify specific protection goals, use patterns, assessment endpoints, and measurement endpoints to address areas of potential concern. When specific protection goals are discussed, they should be concise and actionable with well‐defined assessment and measurement endpoints established in advance of initiating a higher tier study. In addition, to facilitate effective science‐based conversations between registrants and regulatory authorities, it is recommended that adequate background materials be provided from both sides well in advance of meetings. Submitted materials should be manageable and preferably summarized so as to facilitate review by the regulatory agencies in recognition of their tight regulatory timeframes and limited staff resources. If this exchange of information is not completed, with enough time for all parties to review the materials, there will not be an effective dialogue and decision making will be hampered.

The second consensus recommendation addressed the design of higher tier studies so that if and when these studies are deemed appropriate, they can provide high value to the risk assessment and risk management process. When additional data are required, the regulatory authority and registrant should work to prioritize studies that will provide highly relevant findings that address the most significant areas of concern. The nature of the study will depend on several factors such as the results of lower tier studies, environmental fate, and use patterns. It was recommended that the design of higher tier studies should minimize complexity, incorporating higher tier data and assessment procedures using a stepwise process, moving from simple to complex, so that the results are easily interpreted and generalized across a diversity of environments. Another important area of discussion within the group was that methods should be formalized in guidance documents, and protocols for higher tier studies should provide well‐defined performance and acceptability criteria that meet USEPA's Guidance on Systematic Planning Using the Data Quality Objective Process (USEPA 2006). In addition, the design and acceptance of higher tier studies would benefit from the development of standard evaluation procedures for commonly requested higher tier studies. When registrants provide higher tier studies and wish to improve the likelihood of their acceptance and use, registrants should provide a summary describing how the supplementary information informs risk assessment or risk management decisions. In other words, the registrants should explain why the measurement endpoints are important indicators and how they relate to the assessment endpoint and specific protection goal.

**Figure 2:**
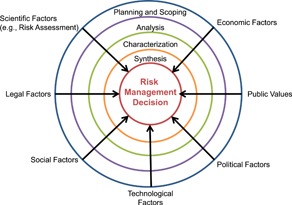
Conceptual diagram describing the USEPA Risk Management Decision Framework. USEPA = United States Environmental Protection Agency.

The third recommendation advocated for greater transparency to understand the process of risk versus benefit decision making and other critical inputs that factor into the current risk management decision framework as shown in Figure 2 (adapted from USEPA 2000). It was recommended that when a risk management decision is made, a summary of the process should be well documented to be a resource for future assessments. In addition, the group agreed that the transparency of the risk assessment and risk management process would benefit from documentation of reviews that were prepared to evaluate higher tier data for relevance and reliability.

As a fourth recommendation, participants felt that it would be beneficial to conduct a retrospective analysis of success–failure learnings from higher tier studies and to highlight where these studies helped to inform risk assessment or risk management decisions. In addition, a postmortem meeting on the pyrethroid risk assessment and risk management process was recommended as a case study to identify what worked and where the process could have been improved regarding acceptance or rejection of higher tier data.

As an outcome of this workshop, a further recommendation is the formation of a multisector workgroup of experts tasked with formulating improved approaches to address the consensus recommendations. The workgroup should perform this task in a transparent manner with clear communication to the regulatory community and the public via follow‐up publications and communications as needed. It is expected that the proposed work will improve the quality and value of higher tier studies submitted to regulatory authorities, will reduce unnecessary higher tier study submissions, and promote a more transparent pesticide risk management process.

## SUPPLEMENTAL DATA

Supplemental Data file contains a list of participants in the workshop summarized by this manuscript.

## Supporting information

This article contains online‐only Supplemental Data.

## References

[ieam4173-bib-0001] Arts GHP , Guijse‐Bogdan LL , Belgers JDM , Van Rhenen‐Kersten CH , Van Wijngaarden RPA , Roessink I , Maund SJ , Van den Brink PJ , Brock TCM . 2006 Ecological impact in ditch mesocosms of simulated spray drift from a crop protection program for potatoes. Integr Environ Assess Manag 2:105–125. 16646380

[ieam4173-bib-0002] Baker JL . 1994. Aquatic dialogue group, pesticide risk assessment and mitigation: Final report of the aquatic dialogue group, May 1993–May 1994. Pensacola (FL): SETAC. 188 p. ISBN: 1880611058, 9781880611050.

[ieam4173-bib-0003] Barnthouse LW . 1992 The role models in ecological risk assessment – A 1990's perspective. Environ Toxicol Chem 11(12):1751–1760.

[ieam4173-bib-0004] Bartell SM , Brain RA , Hendley P , Nair SK . 2013 Modeling the potential effects of atrazine on aquatic communities in midwestern streams. Environ Toxicol Chem 32(10):2402–2411. 24006334 10.1002/etc.2332

[ieam4173-bib-0005] Barton AL . 1994 Ecological risk assessment in the Office‐of‐Pesticide Programs In: Kendall RJ, Lacher TE, editors. Wildlife toxicology and population modeling: Integrated studies of agroecosystems. Boca Raton (FL): CRC p 27–31.

[ieam4173-bib-0006] Bondarenko S , Gan J , Spurlock F . 2008 Solid‐phase microextraction (SPME) methods to measure bioavailable concentrations in sediment In: Gan J, Spurlock F, Hendley P, Weston D, editors. Synthetic pyrethroids: Occurrence and behavior in aquatic environments. Vol. 991 Washington (DC): ACS Book Series p 149–168.

[ieam4173-bib-0007] Brain RA , Perine J , Cooke C , Ellis CB , Harrington P , Lane A , O’Sullivan C , Ledson M . 2017 Evaluating the effects of herbicide drift on nontarget terrestrial plants: A case study with mesotrione. Environ Toxicol Chem 36:2465–2475. 28262983 10.1002/etc.3786

[ieam4173-bib-0008] Brock TCM , Bhatta R , van Wijngaarden RPA , Rico A . 2016 Is the chronic Tier‐1 effect assessment approach for insecticides protective for aquatic ecosystems? Integr Environ Assess Manag 12(4):747–758. 26442690 10.1002/ieam.1719

[ieam4173-bib-0009] Campbell PJ , Arnold D , Brock TCM , Grandy N , Heger W , Heimbach F , Maund SJ , Streloke M . 1998. Guidance document: Higher‐tier aquatic risk assessment for pesticides (HARAP). In: SETAC‐Europe/OECD/EC Workshop; 1998 April 19–22; Lacanau Ocean, France. Brussels (BE): SETAC‐Europe. 61 p.

[ieam4173-bib-0010] Clark JR , Goodman LR , Bothwick PW , Patrick JR , Cripe GM , Moody PM , Moore JC , Lores EM . 1989 Toxicity of pyrethroids to marine invertebrates and fish: A literature review and test results with sediment‐sorbed chemicals. Environ Toxicol Chem 8:393–401.

[ieam4173-bib-0011] Connolly JP . 1985 Predicting single‐species toxicity in natural‐water systems. Environ Toxicol Chem 4(4):573–582.

[ieam4173-bib-0012] Day K . 1991 Effects of dissolved carbon on accumulation and acute toxicity of fenvalerate, deltamethrin and cyhalothrin to *Daphnia magna* (Straus). Environ Toxicol Chem 10:91–101.

[ieam4173-bib-0013] Davidson P , Jones R , Harbourt C , Hendley P , Goodwin G , Sliz B . 2014 Major transport mechanisms of pyrethroids in residential settings and effects of mitigation measures. Environ Toxicol Chem 33:52–60. 24105831 10.1002/etc.2411 PMC4255737

[ieam4173-bib-0014] Di Toro D , Zarba C , Hansen D , Berry W , Swartz R , Cowan C , Pavlou S , Allen H , Thomas N , Paquin P . 1991 Technical basis for establishing sediment quality criteria for nonionic organic chemicals using equilibrium partitioning. Environ Toxicol Chem 10:1541–1583.

[ieam4173-bib-0015] Dixon KR . 2012 A model to predict the effects of insecticides on avian populations In: Dixon KR, editor. Modeling and simulation in ecotoxicology with applications in Matlab(R) and Simulink(R). Boca Raton (FL): CRC p 191–208.

[ieam4173-bib-0016] Dohmen GP , Preuss TG , Hamer M , Galic N , Strauss T , van den Brink PJ , De Laender F , Bopp S . 2016 Population‐level effects and recovery of aquatic invertebrates after multiple applications of an insecticide. Integr Environ Assess Manag 12(1):67–81. 26119989 10.1002/ieam.1676

[ieam4173-bib-0017] [ECOFRAM] Ecological Committee on FIFRA Risk Assessment Methods . 1999 Aquatic draft report. Washington (DC): US Environmental Protection Agency. 451 p. [accessed 2018 June 29]. https://archive.epa.gov/oppefed1/web/pdf/aquareport.pdf

[ieam4173-bib-0019] [EFSA] European Food Safety Authority . 2013 Guidance on tiered risk assessment for plant protection products for aquatic organisms in edge‐of‐field surface waters. EFSA J 11(7):3290 10.2903/j.efsa.2013.3290

[ieam4173-bib-0020] [EFSA] European Food Safety Authority . 2014 Scientific opinion on good modelling practice in the context of mechanistic effect models for risk assessment of plant protection products. EFSA J 12(3):3589 10.2903/j.efsa.2014.3589

[ieam4173-bib-0021] [EFSA] European Food Safety Authority . 2016 Recovery in environmental risk assessments at EFSA. EFSA J 14(2):4313 10.2903/j.efsa.2016.4313 PMC13093211 42016879

[ieam4173-bib-0022] Etterson M , Garber K , Odenkirchen E . 2017 Mechanistic modeling of insecticide risks to breeding birds in North American agroecosystems. PLoS One 12(5):e0176998. 28467479 10.1371/journal.pone.0176998 PMC5415183

[ieam4173-bib-0023] Focks A , ter Horst M , van den Berg E , Baveco H , van den Brink PJ . 2014 Integrating chemical fate and population‐level effect models for pesticides at landscape scale: New options for risk assessment. Ecol Modell 280:102–116.

[ieam4173-bib-0024] Gergs A , Gabsi F , Zenker A , Preuss TG . 2016 Demographic toxicokinetic‐toxicodynamic modeling of lethal effects. Environ Sci Technol 50:6017–6024. 27158745 10.1021/acs.est.6b01113

[ieam4173-bib-0025] Giddings J , Brock T , Heger W , Heimbach F , Maund S , Norman S , Ratte H‐T , Schäfers C , Streloke M . 2002. Community‐level aquatic system studies – Interpretation criteria. In: Proceedings from the CLASSIC Workshop; 30 May–2 June 1999; Schmallenberg, Germany. Pensacola (FL): SETAC. 44 p.

[ieam4173-bib-0026] Giddings J , Solomon K , Maund S . 2001 Probabilistic risk assessment of cotton pyrethroids: II. Aquatic mesocosm and field studies. Environ Toxicol Chem 20:660–668. 11349869 10.1897/1551-5028(2001)020<0660:praocp>2.0.co;2

[ieam4173-bib-0027] Giddings J , Wirtz J , Campana D , Campasino K , Hendley P , Winchell M , Holmes C , Desmarteau D , Ritter A . 2017 Pyrethroid working group response to the EFED preliminary risk assessment (PRA) for pyrethroids and pyrethrins. Valdosta (GA): Pyrethroid Working Group. 548 p. PWG‐PRAresp_02. [accessed 2018 July 3]. https://www.regulations.gov/document?D=EPA-HQ-OPP-2010-0480-0252

[ieam4173-bib-0028] Giddings JM , Wirtz J , Campana D , Dobbs M , Mitchell G . 2016 Derivation of combined species sensitivity distributions for acute toxicity of pyrethroids to aquatic animals. Valdosta (GA): Pyrethroid Working Group. 51 p. PWG‐ERA‐21. [accessed 2018 June 29]. http://pyrethroids.com/wp-content/uploads/2018/04/Pyrethroid-Species-Sensitivity-Distributions-SSDs_PWG.pdf

[ieam4173-bib-0029] Grimm V , Augusiak J , Focks A , Frank BM , Gabsi F , Johnston ASA , Liu C , Martin BT , Meli M , Radchuk V et al. 2014 Towards better modelling and decision support: Documenting model development, testing, and analysis using TRACE. Ecol Modell 280:129–139.

[ieam4173-bib-0030] Hamer M , Goggin U , Muller K , Maund S . 1999 Bioavailability of lambda‐cyhalothrin to *Chironomus riparius* in sediment‐water and water‐only systems. Aquat Ecosyst Health Manage 2:403–412.

[ieam4173-bib-0031] Hamer MJ , Maund SJ , Hill IR . 1992. Laboratory methods for evaluating the impact of pesticides on water/sediment organisms. In: Proceedings Brighton Crop Protection Conference–Pests and Diseases; 1992 Nov 23–26; Brighton, UK. Vol 2. Farnham (UK): BCPC. p 487–496.

[ieam4173-bib-0032] Hanson N , Stark JD . 2012 Comparison of population level and individual level endpoints to evaluate ecological risk of chemicals. Environ Sci Technol 46(10):5590–5598. 22509961 10.1021/es3008968

[ieam4173-bib-0034] Hilbers JP , Hoondert RPJ , Schipper AM , Huijbregts MAJ . 2018 Using field data to quantify chemical impacts on wildlife population viability. Ecol Appl 28(3):771–785. 29336512 10.1002/eap.1685

[ieam4173-bib-0035] Hommen U , Forbes V , Grimm V , Preuss TG , Thorbeck P , Ducrot V . 2016 How to use mechanistic effect models in environmental risk assessment of pesticides: Case studies and recommendations from the SETAC workshop MODELINK. Integr Environ Assess Manag 12(1):21–31. 26437629 10.1002/ieam.1704

[ieam4173-bib-0036] Hunter W , Yang W , Spurlock R , Gan J . 2008 Solid‐phase microextraction (SPME) methods to measure bioavailable concentrations in surface waters In: Gan J, Spurlock F, Hendley P, Weston D, editors. Synthetic pyrethroids: Occurrence and behavior in aquatic environments. Vol 991 Washington (DC): ACS Book Series p 130–148.

[ieam4173-bib-0037] Jackson S , Winchell M . 2011 Use of the EPA storm water management model (SWMM) for diagnosis of pesticide off‐target movement from residential areas In: Bret B, Potter T, Gan J, editors. Pesticide mitigation strategies for surface water quality. Vol 1075 Washington (DC): ACS p 309–327.

[ieam4173-bib-0085] Kendall RJ , Lacher TE . 1993 Wildlife toxicology and population modeling: Integrated studies of agroecosystems. SETAC Special Publications Series. Boca Raton (FL): CRC Pr. 592 p.

[ieam4173-bib-0038] King RS , Brain RA , Back JA , Becker C , Wright MV , Djomte VT , Scott WC , Virgil SR , Brooks BW , Hosmer AJ et al. 2016 Effects of pulsed atrazine exposures on autotrophic community structure, biomass, and production in field‐based stream mesocosms. Environ Toxicol Chem 35:660–675. 26292195 10.1002/etc.3213

[ieam4173-bib-0040] Kohler HR , Triebskorn R . 2013 Wildlife ecotoxicology of pesticides: Can we track effects to the population level and beyond? Science 341(6147):759–765. 23950533 10.1126/science.1237591

[ieam4173-bib-0041] Lari L , Massi A , Fossi MC , Casini S , Leonzio C , Focardi S . 1994 Evaluation of toxic effects of the organophosphorus insecticide azinphos‐methyl in experimentally and naturally exposed birds. Arch Environ Contam Toxicol 26(2):234–239.

[ieam4173-bib-0042] Madrigal JL , Pixton GC , Collings BJ , Booth GM , Smith HD . 1996 A comparison of two methods of estimating bird mortalities from field‐applied pesticides. Environ Toxicol Chem 15(6):878–885.

[ieam4173-bib-0043] Mancini JL . 1983 A method for calculating effects, on aquatic organisms, of time‐varying concentrations. Water Res 10:1355–1362.

[ieam4173-bib-0044] Maund S , Hamer M , Lane M , Farrelly E , Rapley J , Goggin U , Gentle W . 2002 Partitioning, bioavailability, and toxicity of the pyrethroid insecticide cypermethrin in sediments. Environ Toxicol Chem 21:9–15. 11808535

[ieam4173-bib-0045] Maund SJ , Hamer MJ , Warinton JS , Kedwards TJ . 1998 Aquatic ecotoxicology of the pyrethroid insecticide lamda‐cyhalothrin: Considerations for higher‐tier aquatic risk assessment. Pestic Sci 54:408–417.

[ieam4173-bib-0046] McConnell L , Samel A . 2018 Second workshop on innovation and regulation in agriculture addresses the challenges of higher‐tier data. SETAC Globe 19(2). [accessed 3 Aug 2018]. https://globe.setac.org/2017-workshop-innovation/

[ieam4173-bib-0047] [NRC] National Research Council . 2013 Assessing risks to endangered and threatened species from pesticides. Committee on Ecological Risk Assessment under FIFRA and ESA. Washington (DC): Natl Academies 194 p. 10.17226/18344

[ieam4173-bib-0048] [NRC] National Research Council . 2017 Preparing for future products of biotechnology. National Academies of Sciences, Engineering, and Medicine. Washington (DC): Natl Academies 230 p. 10.17226/24605 28737846

[ieam4173-bib-0049] Overmyer J , Feken M , Ruddle N , Bocksch S , Hill M , Thompson H . 2018 Thiamethoxam honey bee colony feeding study: Linking effects at the level of the individual to those at the colony level. Environ Toxicol Chem 37:816–828. 29265500 10.1002/etc.4018

[ieam4173-bib-0050] Pastorok RA , Akcakaya HS . 2002 Methods. Chapter 2 In: Pastorok RA, Bartell SM, Ferson S, Ginzburg LR, editors. Ecological modeling in risk assessment, chemical effects on populations, ecosystems and landscapes. Boca Raton (FL): CRC p 22–34.

[ieam4173-bib-0051] Posthuma L , Suter G , Traas T , editors. 2001 Species sensitivity distributions in ecotoxicology. Boca Raton (FL): CRC. 587 p.

[ieam4173-bib-0052] Purucker ST , Welsh CJE , Stewart RN , Starzec P . 2007 Use of habitat‐contamination spatial correlation to determine when to perform a spatially explicit ecological risk assessment. Ecol Modell 204(2):180–192.

[ieam4173-bib-0053] Schmolke A , Thorbek P , Chapman P , Grimm V . 2010 Ecological models and pesticide risk assessment: Current modeling practice. Environ Toxicol Chem 29(4):1006–1012. 20821532 10.1002/etc.120

[ieam4173-bib-0054] Shillabeer N , Smyth DV , Tattersfield L . 2000. Higher tier risk assessment of agrochemicals, incorporating sediment into algal test systems. In: Proceedings Brighton Crop Protection Conference–Pests and Diseases; 1992 Nov 23–26; Brighton, UK. Farnham (UK): BCPC. p 359–364.

[ieam4173-bib-0055] Solomon KR , Baker DB , Richards RP , Richards RP , Dixon KR , Klaine SJ , La Point TW , Kendall RJ , Weisskopf CP , Giddings JM et al. 1996 Ecological risk assessment of atrazine in North American surface waters. Environ Toxicol Chem 15(1):31–74. 10.1002/etc.2050 23147529

[ieam4173-bib-0056] Solomon KR, Brock TCM, de Zwart D, Dyer SD, Posthuma L, Richards SM, Sanderson H, Sibley PK, van den Brink PJ, editors. 2008 Extrapolation practice for ecotoxicological effect characterization of chemicals. Boca Raton (FL): CRC. 380 p.

[ieam4173-bib-0057] Solomon KR , Giesy JP , Kendall RJ , Best LB , Coats JR , Dixon KR , Hooper MJ , Kenaga EE , McMurry ST . 2001 Chlorpyrifos: Ecotoxicological risk assessment for birds and mammals in corn agroecosystems. Hum Ecol Risk Assess 7(3):497–632.

[ieam4173-bib-0076] Teske ME , Bird SL , Esterly DM , Curbishley TB , Ray SL , Perry SG . 2002 AgDRIFT: A model for estimating near‐field spray drift from aerial applications. Environ Toxicol Chem 21:659–671. 11878480 10.1897/1551-5028(2002)021<0659:aamfen>2.0.co;2

[ieam4173-bib-0058] Topping CJ , Craig PS , de Jong F , Klein M , Laskowski R , Manachini B , Pieper S , Smith R , Sousa JP , Streiss F et al. 2015 Towards a landscape scale management of pesticides: ERA using changes in modelled occupancy and abundance to assess long‐term population impacts of pesticides. Sci Total Environ 537:159–169. 26318547 10.1016/j.scitotenv.2015.07.152

[ieam4173-bib-0059] [USEPA] United States Environmental Protection Agency . 1998. Guidance for ecological risk assessment. Washington (DC). 188 p. EPA/630/R‐95/002F.

[ieam4173-bib-0060] [USEPA] United States Environmental Protection Agency . 2000. Science Policy Council handbook. Washington (DC): Office of Science Policy, Office of Research and Development. 53 p. EPA 100‐B‐00‐002.

[ieam4173-bib-0061] [USEPA] United States Environmental Protection Agency . 2004 Overview of the ecological risk assessment process in the Office of Pesticide Programs, U.S. Environmental Protection Agency: Endangered and threatened species effects determinations. Washington (DC): Office of Prevention, Pesticides and Toxic Substances, Office of Pesticide Programs. 92 p. [accessed 2018 Jun 29]. https://www.epa.gov/sites/production/files/2014-11/documents/ecorisk-overview.pdf

[ieam4173-bib-0062] [USEPA] United States Environmental Protection Agency . 2006. Guidance on systematic planning using the data quality objective process. Washington (DC). 121 p. EPA QA/G‐4. EPA/240/B‐06/001.

[ieam4173-bib-0063] [USEPA] United States Environmental Protection Agency . 2009. Alkyl amine polyalkoxylates (JITF CST 4 Inerts Ingredients). Human health risk assessment to support proposed exemption from the requirement of a tolerance when used as inert ingredients in pesticide formulations. Washington (DC). 94 p.

[ieam4173-bib-0064] [USEPA] United States Environmental Protection Agency . 2010 Clothianidin registration of Prosper T400 seed treatment on mustard seed (oilseed and condiment) and Poncho/Votivo seed treatment on cotton. Washington (DC). 100 p. [accessed 2018 Jun 29]. https://archive.epa.gov/pesticides/chemicalsearch/chemical/foia/web/pdf/044309/044309-2010-11-02b.pdf

[ieam4173-bib-0065] [USEPA] United States Environmental Protection Agency . 2012. Ecological effects test guidelines OCSPP 850.4150: Vegetative vigor. Washington (DC): Office of Chemical Safety and Pollution Prevention. 22 p. EPA 712‐C‐001.

[ieam4173-bib-0066] [USEPA] United States Environmental Protection Agency . 2016a Refinements for risk assessment of pesticide treated seed – Interim guidance. Washington (DC). 12 p. [accessed 2018 Jun 29]. https://www.epa.gov/sites/production/files/2016-04/documents/interimseedtreatmentguidance2016.pdf

[ieam4173-bib-0067] [USEPA] United States Environmental Protection Agency . 2016b. Tioxazafen (MON 102100): Ecological risk assessment for use as a seed treatment on corn, soybean, and cotton. Washington (DC). 60 p. EPA‐HQ‐OPP‐2015‐0215‐0010.

[ieam4173-bib-0068] [USEPA] United States Environmental Protection Agency . 2016c Pesticide in water calculator version 1.50 and 1.52 user manual. Washington (DC). [accessed 2018 Jun 22]. 23 p. https://www.epa.gov/pesticide-science-and-assessing-pesticide-risks/pesticide-water-calculator-version-150-and-152-user

[ieam4173-bib-0069] [USEPA] United States Environmental Protection Agency . 2016d. Preliminary comparative environmental fate and ecological risk assessment for the registration review of eight synthetic pyrethroids and the pyrethrins. Washington (DC). 800 p. EPA‐HQ‐OPP‐2010‐0384‐0045.

[ieam4173-bib-0070] [USEPA] United States Environmental Protection Agency , [PMRA] Health Canada Pest Management Regulatory Authority , [CADPR] California Department of Pesticide Regulation . 2014. Guidance for assessing pesticide risks to bees. July 19, 2014. Washington (DC). 59 p.

[ieam4173-bib-0071] Van den Brink PJ . 2013 Assessing aquatic population and community‐level risks of pesticides. Environ Toxicol Chem 32(5):972–973. 23589418 10.1002/etc.2210

[ieam4173-bib-0072] Van Wijngaarden RPA , Brock TCM , Douglas MT . 2005 Effects of chlorpyrifos in freshwater model ecosystems: The influence of experimental conditions on ecotoxicological thresholds. Pest Manage Sci 61(10):923–935. 10.1002/ps.1084 15962350

[ieam4173-bib-0073] Wang M . 2013 From home range dynamics to population cycles: Validation and realism of a common vole population model for pesticide risk assessment. Integr Environ Assess Manag 9(2):294–307. 23086922 10.1002/ieam.1377

[ieam4173-bib-0075] Yang W , Spurlock F , Liu W , Gan J . 2006 Effects of dissolved organic matter on permethrin bioavailability to *Daphnia* species. J Agric Food Chem 54:3967–3972. 16719522 10.1021/jf060217y

