# Supplementary material for: Overcoming Challenges of Incorporating Higher Tier Data in Ecological Risk Assessments and Risk Management of Pesticides in the United States: Findings and Recommendations from the 2017 Workshop on Regulation and Innovation in Agriculture
Source: Integr Environ Assess Manag. 2019 Aug 7;15(5):714–25. doi: 10.1002/ieam.4173 (PMC6852661; doi:10.1002/ieam.4173)
Supplement: Supplementary file 1 — This article contains online‐only Supplemental Data. [file IEAM-15-714-s001.docx]

**Overcoming Challenges of Incorporating Higher-Tier Data in Ecological Risk Assessments and Risk Management of Pesticides in the United States: Findings and Recommendations from the 2017 Workshop on Regulation and Innovation in Agriculture**

**Supplemental Materials**

**List of Participants and affiliations listed at the time of the workshop**

**Organizing Committee**

Linda Abbott, USDA Office of Risk Assessment and Cost Benefit Analysis

George Cobb, Baylor University

Kevin Costello, US EPA, Office of Pesticide Programs

Jeffrey Giddings, Compliance Services International

Matt Kern, Waterborne Environmental, Inc.

Laura McConnell (chair), Bayer CropScience

Edward Odenkirchen, US EPA, Office of Pesticide Programs

Danesha Seth-Carley (co-chair), NC State Southern IPM Center

**Participants**

Kevin Armbrust, Louisiana State University

Teung Chin, USDA Office of Pest Management Policy

W. Gregory Cope, NC State

Marie Delorenzo, NOAA NCCOS

Jean-Jacques Dubois, NC State

Dave Fischer, Bayer CropScience

Scott Jackson, Valent U.S.A. Corporation

Spencer Mortensen, BASF Corporation

Alan Samel, DuPont

Tamar Schlekat, SETAC

Geoff Scott, University of South Carolina

Christian Schlekat, NiPERA

Keith Solomon, University of Guelph (Canada)

Claire Terry, Dow AgroSciences

Garland Waleko, US EPA, Office of Pesticide Programs

Theodore Valenti, Syngenta Crop Protection
